# A New Nitroimidazole-Based Drug Attenuates Skeletal Myositis and Overcomes Benznidazole Resistance in Experimental Chagas Disease

**DOI:** 10.1021/acsptsci.6c00179

**Published:** 2026-06-02

**Authors:** Graziela Domingues De Almeida Lima, Elda Gonçalves-Santos, Monique Dias Benedetti, Thiago Donizeth Silva, Aristides Pinheiro, Silvânia Mól Pelinsari, Luis Felipe Cunha dos Reis, Reggiani Vilela Gonçalves, Ivo Santana Caldas, Lucas Lopardi Franco, Rômulo Dias Novaes

**Affiliations:** † Programa de Pós-Graduação em Ciências Biológicas, 74347 Universidade Federal de Alfenas, Alfenas, State of Minas Gerais 37130-001, Brazil; ‡ Programa de Pós-Graduação em Biociências Aplicadas à Saúde, Universidade Federal de Alfenas, Alfenas, State of Minas Gerais 37130-001, Brazil; § Departamento de Biologia Geral, Programa de Pós-Graduação em Biologia Celular e Estrutural, Universidade Federal de Viçosa, Viçosa, State of Minas Gerais 36570-900, Brazil; ∥ Instituto de Ciências Biomédicas, Universidade Federal de Alfenas, Alfenas, State of Minas Gerais 37130-001, Brazil; ⊥ Departamento de Biologia Animal, Programa de Pós-Graduação em Biologia Animal, Universidade Federal de Viçosa, Viçosa, State of Minas Gerais 36570-900, Brazil; # North Carolina State University (NCSU), Kannapolis, North Carolina 28081, United States; ¶ Instituto de Ciências Farmacêuticas, Laboratório de Pesquisa em Química Farmacêutica (LQFar), Universidade Federal de Alfenas, Alfenas, State of Minas Gerais 37130-001, Brazil

**Keywords:** antiparasitic chemotherapy, chagas disease, oxidative stress, parasitology, skeletal muscle

## Abstract

Skeletal myositis is a disabling complication of Chagas disease for which the first-choice antiparasitic chemotherapy has limited efficacy. Therefore, we synthesized and investigated the potential of the novel nitroimidazole-based drug 1-(2-(2-methoxy-6-nitro-4-propylphenoxy)­ethyl)-2-methyl-5-nitro-1*H*-imidazole (ME) administered alone or combined with benznidazole (BZ) to control myocytes parasitism and skeletal myositis in Chagas disease. ME antiparasitic efficacy was investigated in vitro and in vivo in C2C12 skeletal myocytes and mice challenged with *Trypanosoma cruzi* Y strain. Physicochemical modeling indicated that ME presented characteristics compatible with orally bioactive drugs. Similar to BZ, our results indicated that ME showed a marked antiparasitic effect in vitro, attenuating *T. cruzi* viability, infection rate, and parasite load in C2C12 myocytes in a dose-dependent manner. In vivo, *T. cruzi* infection induced intense parasitemia, muscle parasitism, oxidative stress, and inflammation, features associated with pathological microstructural remodeling of the skeletal muscle. ME administered alone and mainly in combination with BZ significantly reduced parasitemia, parasite load, production of reactive oxygen species (ROS) and nitrogen species (RNS), oxidation of lipids and cellular proteins, inflammation (e.g., inflammatory infiltrate, NAG and MPO activity, IFN-γ, TNF, IL-6, and IL-10 levels), and microstructural damage in skeletal muscle of *T. cruzi*-infected animals. Our findings provide evidence that ME has direct antiparasitic effects in vitro and in vivo, showing potential efficacy for use as monotherapy and mainly in combination with BZ. This combination may be relevant to improve the etiological treatment of Chagas disease, simultaneously attenuating parasitism, oxidative stress, and skeletal myositis more efficiently than monotherapy with these drugs.

## Graphical abstract

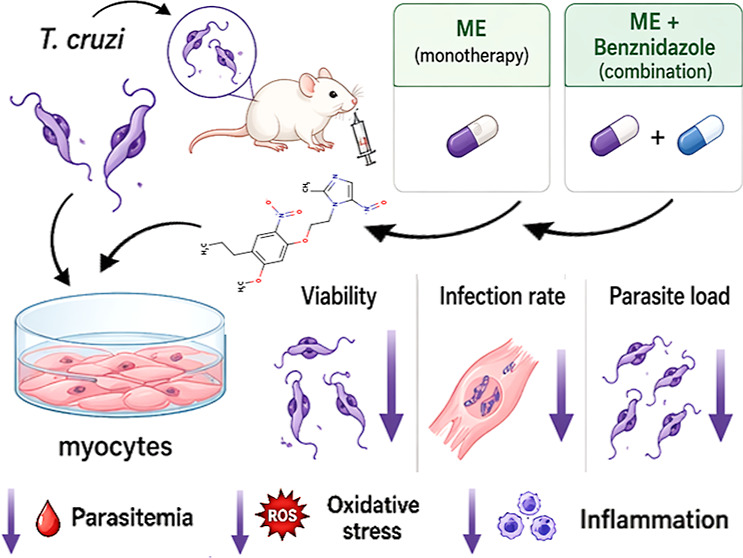

Chagas disease (ChD) is a neglected systemic infection caused by the parasite *Trypanosoma cruzi*.^1^ It is endemic to Latin America, and its prevalence is rising in nonendemic areas such as North America and Europe, especially due to migratory flows of infected people, vertical transmission, and transplantation of *T. cruzi*-infected tissues and organs.^2,3^ ChD primarily affects poor communities with limited access to early diagnosis and etiological treatment in the acute phase of the disease, a period in which parasitological cure rates (60%–75%) are more favorable.^4,5^ Accordingly, infected patients are predominantly diagnosed in the chronic phase of ChD, at which point antiparasitic chemotherapy has low efficacy (cure rate between 0% and 10%).^1,5^

Chronic symptomatic ChD is associated with high morbidity and mortality and is characterized by systemic parasitism and inflammation, especially in immune and muscle cells and organs.^6^ Due to the marked myotropism exhibited by most *T. cruzi* clones, the heart and skeletal muscles are priority targets of the parasite, constituting important reservoirs that contribute to perpetuating the infection.^7^ There is consistent evidence that skeletal muscle parasitism is associated with intense oxy-inflammation, myonecrosis, myocytolysis, and neuromuscular degeneration,^8^ pathological events linked to extensive loss of muscle parenchyma, progressive fibrosis, and contractile dysfunction.^9,10^ Despite the severity of skeletal muscle infection and unlike Chagas myocarditis, the relevance of standard antiparasitic chemotherapy and the development of innovative trypanocidal drugs for *T. cruzi*-induced skeletal myositis treatment remains overlooked.

Currently, specific ChD treatment is restricted to the nitrocompounds nifurtimox (NFx) and benznidazole (Bz).^1,5^ Due to high systemic toxicity, NFx has been discontinued in most endemic countries.^1,11^ Thus, Bz has become the first-line drug for ChD treatment, being listed as an essential medicine by the World Health Organization.^11^ However, Bz is also toxic and causes serious side effects (e.g., bone marrow depression, peripheral neuropathy, dermatitis, and digestive intolerance), which often lead to treatment discontinuation and therapeutic failure.^5,11^ In addition to these characteristics, the emergence of parasites resistant to reference chemotherapy, problems with solubility and bioavailability help to explain the limited Bz effectiveness in ChD treatment.^12^

Considering *T. cruzi* infection severity and parasite resistance to the reference drugs currently available, the development of more efficient chemotherapeutic strategies represents the most urgent challenge to combat ChD.^10,11^ The synthesis of new chemical entities from the complexation of molecules with different and complementary antiparasitic properties, as well as combination chemotherapy protocols, has emerged as a rational opportunity to overcome parasite resistance, protect target organs from *T. cruzi* infection, and increase cure rates.^1,5,13^ Based on the trypanocidal potential identified in vitro for the drugs 2-(2-methyl-5-nitro-1*H*-imidazole-1-yl)­ethanol and allylguaiacol,^14^ we synthesized the innovative molecular compound 1-(2-(2-methoxy-6-nitro-4-propylphenoxy)­ethyl)-2-methyl-5-nitro-1*H*-imidazole (ME), whose therapeutic potential needs to be determined. Therefore, we combined physicochemical and ADME (absorption, distribution, metabolism, and excretion) modeling, in vitro, and in vivo methods to investigate the effectiveness of this novel nitroimidazole-based drug in controlling parasitemia, myocyte parasitism, and skeletal muscle oxy-inflammation when administered alone or combined with benznidazole (BZ) in ChD.

## Experimental Section

### Chemical Synthesis of the Novel Nitroimidazole-Based Drug 1-(2-(2-Methoxy-6-nitro-4-propylphenoxy)­ethyl)-2-methyl-5-nitro-1*H*-imidazole

The chemical synthesis protocol was developed as previously reported.^13^ Briefly, the molecule 2-methyl-5-nitroimidazole-1-ethanol was converted into its mesylated derivative (1) through a classical reaction with methanesulfonyl chloride in pyridine at 0 °C. In parallel, 4-propyl guaiacol was reacted with KHSO_4_ and NaNO_3_ under magnetic stirring in a dichloromethane and silica-gel suspension at room temperature, to obtain 6-nitro-4-propyl guaiacol (2). The coupling of 1 and 2 was carried out through a classical S_N_2 reaction, where the phenol derivative (2) was dissolved in *N*,*N*-dimethylformamide in the presence of sodium bicarbonate at 80 °C, and 1 was added slowly. The mixture was kept under stirring and heating for 12 h, and the resulting crude product (1-(2-(2-methoxy-6-nitro-4-propylphenoxy)­ethyl)-2-methyl-5-nitro-1*H*-imidazole) was purified by column chromatography (hexane 7–3 ethyl acetate), with 85% yield. The synthesis scheme is presented in Figure 1. The purified molecule (nitroimidazole-based drug) was chemically characterized using ^1^H nuclear magnetic resonance and ^3^C nuclear magnetic resonance spectroscopy (Figure 2), together with liquid chromatography–mass spectrometry (LC–MS ESI/Q-TOF) (Figure 3).

**Figure 1:**
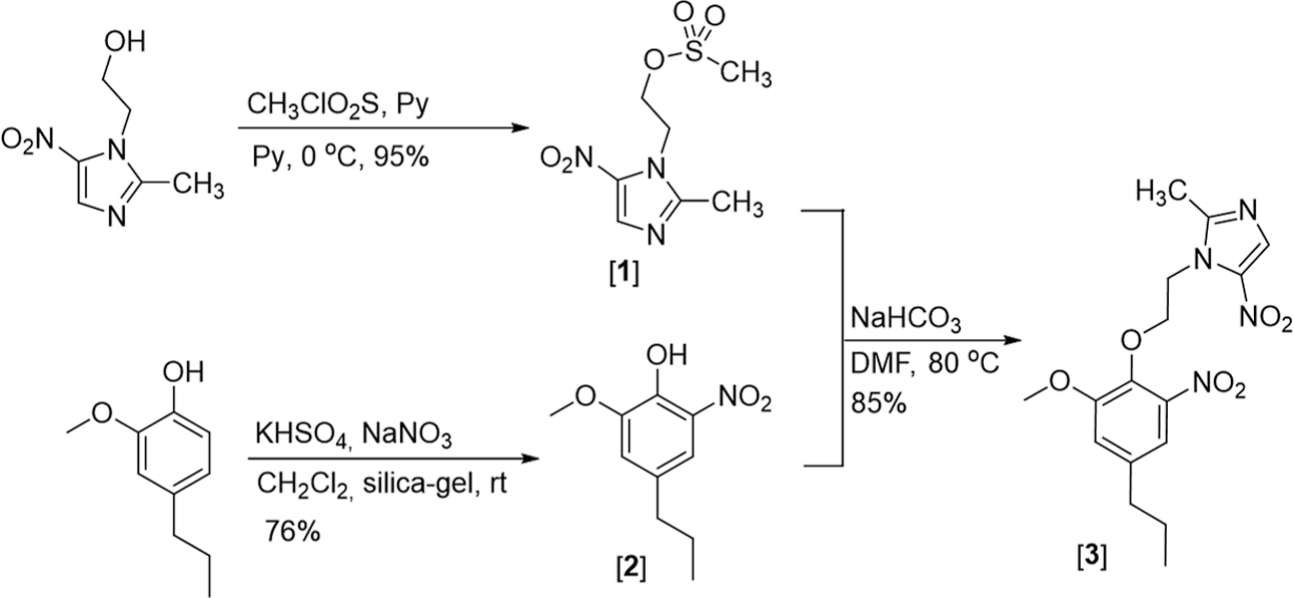
Chemical reactions used to synthesize the novel nitroimidazole-based drug 1-(2-(2-methoxy-6-nitro-4-propylphenoxy)­ethyl)-2-methyl-5-nitro-1*H*-imidazole [3] investigated alone and combined with benznidazole against *T. cruzi* in vitro and in vivo. [1] 2-Methyl-5-nitroimidazole-1-ethanol and [2] 4-propyl guaiacol.

**Figure 2:**
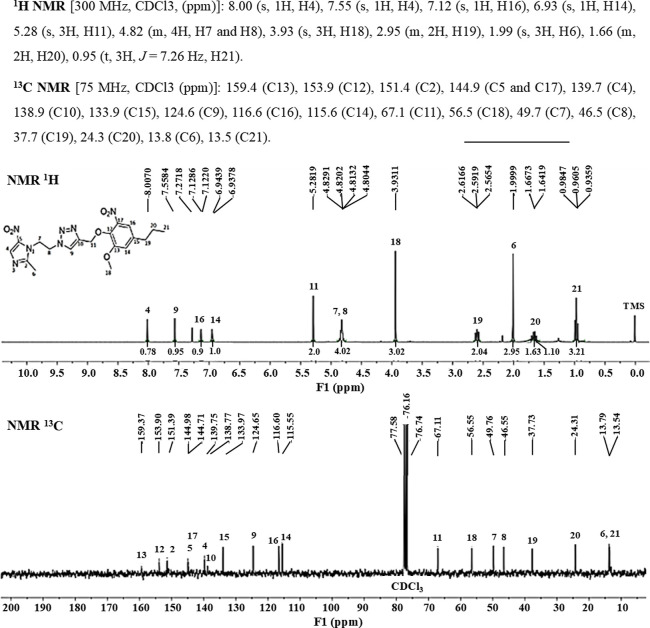
Chemical characterization of the novel nitroimidazole-based drug 1-(2-(2-methoxy-6-nitro-4-propylphenoxy)­ethyl)-2-methyl-5-nitro-1*H*-imidazole (ME) by nuclear magnetic resonance (^1^H NMR).

**Figure 3:**
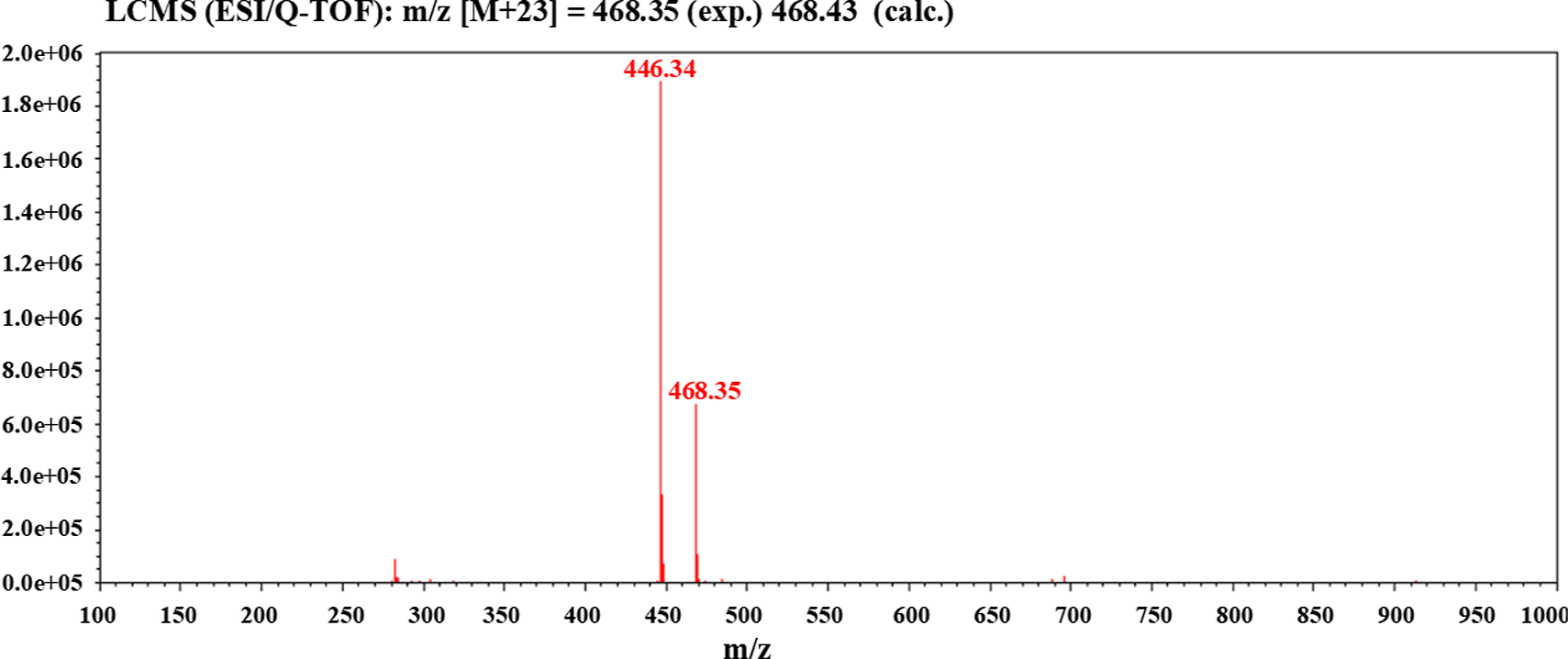
Liquid chromatography–mass spectrometry (LCMS ESI/Q-TOF) profile of the novel nitroimidazole-based drug 1-(2-(2-methoxy-6-nitro-4-propylphenoxy)­ethyl)-2-methyl-5-nitro-1*H*-imidazole (ME).

### Physicochemical, Pharmacokinetic, and Pharmacodynamic Modeling

Physicochemical, pharmacodynamic, and pharmacokinetic parameters were modeled for ME and compared with the same parameters predicted for Bz. Physicochemical information was obtained using the SwissADME server as previously reported.^15^ The Bioavailability Radar was used to estimate molecular characteristics admitting value ranges for drugs with favorable oral bioavailability as follows: (i) lipophilicity (XLOGP3 −0.7 to +5.0), (ii) size (molecular weight 150 to 500 g/mol), (iii) polarity (TPSA 20 to 130 Å^2^), (iv) solubility (log­(*S*) ≤ 6), (v) instauration (fraction of carbons in the sp^3^ hybridization ≥0.25), and (vi) flexibility (≤9 rotatable bonds).

Pharmacokinetic and pharmacodynamic estimates were obtained using the Deep-PK server as previously reported.^16^ Drug permeability was based on Madin–Darby Canine Kidney (MDCK) cell and Caco-2 cell permeability. Results >0.90 indicate high permeability in Caco-2 cells, and can be classified as low (<4 cm/s), moderate (4–70 cm/s), or high (>70 cm/s) in MDCK cells. Intestinal absorption (%) and 50% oral bioavailability were used to estimate the relative intestinal absorption of the oral dose. Drug interaction with P-glycoprotein was investigated to estimate substrate or inhibitor potential, given its impact in efflux-mediated reduction of effective permeability.^16^ The following physicochemical descriptors known to influence diffusion across membranes were analyzed: log *D* at pH 7.4, log *P*, log *S*, p*K*_a_ (acidic and basic), molecular weight, topological polar surface area (TPSA), number of hydrogen bond donors and acceptors, and number of rotatable bonds. Log *P* <0 and TPSA ≥90–120 Å^2^ are markers of limited passive permeability.^16^

### Antiparasitic Activity against Extracellular *T. cruzi* Forms In Vitro

Blood trypomastigotes (Y strain) were isolated from C57BL/6 mice and resuspended in DMEM (Invitrogen, Carlsbad, CA, USA) supplemented with 10% fetal bovine serum (FBS) as previously recommended.^17^ Isolated trypomastigotes (500,000/mL) were incubated for 24 h at 37 °C in RPMI in the absence or presence of different concentrations of ME and Bz dissolved in culture medium in serial dilutions ranging from 1.56 to 100 μg/mL. After the incubation period, the number of live trypomastigotes was microscopically quantified using a Neubauer chamber,^18^ and the IC_50%_ (drug concentration required to inhibit the survival of a parasite population by 50% in vitro) was calculated using the CompuSyn software (ComboSyn Inc., Paramus, NJ, USA) as previously reported.^19^ Accordingly, the 2 doses below and the 3 doses above the IC_50%_ identified for each investigated drug were selected to express their antiparasitic effect.

### Antiparasitic Activity against Intracellular *T. cruzi* Forms In Vitro

C2C12 skeletal myocytes (10,000 cells/well) were incubated in DMEM supplemented with 1% penicillin–streptomycin and 5% FBS for 24 h at 37 °C and 5% CO_2_.^20^ Cell incubation was conducted in 24-well polystyrene plates coated with glass coverslips. Cells were cocultured with trypomastigotes (Y strain) in a 10:1 (parasite/C2C12 cells) ratio. Non internalized parasites were removed 2 h after trypomastigote challenge by washing the cells with fresh DMEM, and more 48 h incubation was used to establish infection.^14^ Then, ME and Bz were added at different concentrations ranging from 0.75 μg/mL to 10 μg/mL, and untreated cells were used as a control. After 48 h incubation with both drugs, the coverslips were fixed in methanol and submitted to the Giemsa-stained method.^14^ Cell infection rate and parasite load were, respectively, determined by counting the number of infected cells and the number of intracellular parasites in 100 cells randomly sampled at ×100 magnification using a light microscope.^14^ The same ME and Bz concentrations referenced by the IC_50%_ against trypomastigotes were investigated in this assay.

### Animals, Infection, and Ethics

Female C57BL/6 mice (8 weeks old and 33.82 ± 3.05 g) were kept in facilities with photoperiod (12 h/12 h light/dark cycles), air humidity (60–70%), and temperature (20 ± 2 °C) controlled.^21^ The animals had free access to chow and water. We used a *T. cruzi* infection model based on intraperitoneal inoculation of 2000 trypomastigotes as previously reported.^9,22,23^ The Y strain was used due to its partial resistance to Bz, high pathogenicity, and infective potential.^22,25^ The infection was confirmed 5 days after *T. cruzi* inoculation by microscopic observation of trypomastigotes in 5 μL blood samples collected from the animals’ tails.^26^ All in vivo protocols were conducted for 15 days to avoid excessive and unnecessary organic debility, respecting ethical-humanitarian ending.^36^ Three independent researchers blinded to the intervention groups administered the treatments, collected the experimental data, and analyzed the research outcomes. The Institutional Ethics Committee for Animal Research approved all procedures involved in the experimental protocol (0010/2022).

### Dose Selection for ME Administration In Vivo

The best ME dose to be used in monotherapy and combined chemotherapy was defined from parasitemia and toxicity of biochemical markers. In this preliminary evaluation, the animals were numerically codified and randomized into 7 groups with 5 animals per group using the random function of the Excel software, as follows: uninfected (UN), infected untreated (INF), and treated daily with ME at 10 (ME1), 50 (ME2), 100 (ME3), 200 (ME4), and 400 (ME5) mg/kg of body weight (mpk). ME was suspended in sterile drinking water and administered by gavage for 10 consecutive days, starting 5 days after *T. cruzi* inoculation (infection confirmation).

Parasitemia was classically assessed by daily counting of circulating trypomastigotes in fresh blood samples examined using a bright-field microscope as previously reported.^27^ Mean parasitemia and peak parasitemia were calculated.^26^ The animals were intraperitoneally anesthetised with ketamine and xylazine (150 and 16 mg/kg, respectively) 24 h after the last treatment. The animals were euthanized by exsanguination, blood samples were centrifuged (3000*g*, 4 °C and 15 min), and serum was used for biochemical analysis of drug toxicity markers. Accordingly, alanine aminotransferase (ALT) and aspartate aminotransferase (AST), creatinine, and urea were automatically quantified in the LabmaxPlenno device following the diagnostic kits guidelines (Labtest, Lagoa Santa, MG, Brazil).

### Monotherapy and Combination Chemotherapy

The effective ME dose for combination chemotherapy was defined as 100 mpk, since it was able to induce the most prominent antiparasitic effect without significant changes in biochemical toxicity markers. Then, female C57BL/6 mice (8 weeks old and 32.27 ± 3.19 g) were randomized into 6 groups with 10 animals per group using the random function of the Excel software, as follows: UN = uninfected, INF = infected untreated, ME = infected treated with 100 mpk ME, BZ = infected treated with 100 mpk Bz, ME1 + BZ = infected treated with 50 mpk ME + 100 mpk Bz, and ME2 + BZ = infected treated with 100 mpk ME + 100 mpk Bz. As previously recommended,^9,25,28^ a concentration 50% lower (here 50 mpk ME) than the effective dose was also included in the therapeutic regimen, considering that the toxicological and antiparasitic effect of combination chemotherapy may be potentiated from synergistic or additive interactions between the associated drugs. The sample size (10 animals per group) was based on preclinical studies focused on combination chemotherapy^22,28^ and calculated considering the statistically significant number of animals corrected for the mortality risk potentially associated with *T. cruzi* infection, as previously reported.^14,29^

The same *T. cruzi* infection model as reported above was used. The benzimidazole dose used here (100 mpk) was selected since it is recognized as the reference treatment in preclinical studies of new trypanocidal drugs.^23,24,30^ This dose corresponds to the recommended dose for human adults (7 mg/kg/day).^31^ Infected and uninfected untreated control mice received water by gavage.^26^ The animals were followed for 15 days post-*T. cruzi* inoculation, and the treatments were administered daily by gavage starting 5 days postinoculation (infection confirmation).

### Skeletal Muscle Parasitism Assay

Twenty-four hours after the last treatment, the animals were anesthetized (16 mg/kg xylazine and 150 mg/kg ketamine) and euthanized by exsanguination. Blood and skeletal muscle (gastrocnemius) were collected. Skeletal muscle parasitism was determined by real-time quantitative PCR (qPCR) according to previous recommendations.^28,32^ In this assay, genomic DNA was extracted from 50 mg gastrocnemius samples using a commercial kit and the manufacturer’s instructions (Promega, São Paulo SP, Brazil). DNA samples were standardized in 25 ng/μL, and PCR reactions were adjusted to 10 μL volume containing 0.50 μM TNF primers or 0.35 μM *T. cruzi* DNA primers, 5 μL SYBR Green (Applied Biosystems, CA, USA), and 50 ng isolated DNA. The primers used were: (a) *T. cruzi* repetitive DNA—reverse (5′-CCAAGCAGCGGATAGTTCAGG-3′) and forward (5′-GCTCTTGCCCACAMGGGTGC-3′) and (b) TNF-α—reverse (5′-CAGCAAGCATCTATGCACTTAGACCCC-3′) and forward (5′-TCCCTCTCATCAGTTCTATGGCCCA-3′).^9^ The reactions were performed in 96-well plates, and a control standard curve containing *T. cruzi*-specific primers and mice-specific primers without DNA and with DNA from uninfected mice was produced.^28^ Parasite load (PaL), indicated as *T. cruzi* DNA levels, was normalized according to TNF results as previously reported.^32^

### Reactive Oxygen Species and Nitric Oxide Assay

Gastrocnemius samples were frozen in liquid nitrogen and homogenized in lysis buffer (50 mM Tris [pH 7.5], 2% Triton X-100, 1 mM phenylmethanesulfonyl fluoride, 40 mM HEPES, and 1 mM EDTA). The homogenate was sonicated on ice for 1 min and centrifuged at 4 °C for 10 min at 8000*g*. Total reactive oxygen species (ROS) were quantified in 150 μL supernatant samples using a CM-H2DCFDA-based biochemical kit and the manufacturer’s instructions (Waltham, Massachusetts, USA). Total pro-oxidants were quantified by spectrofluorimetry at 485 nm/520 nm excitation/emission.^21^

Nitric oxide was estimated using the Griess method using a 96-wells colorimetric kit and the manufacturer’s instructions (Nitric Oxide Assay [EMNSO], Thermo Fisher Scientific, Waltham, MA, USA) as previously reported.^9^ In this assay, the enzyme nitrate reductase was used to catalyze nitrate conversion to nitrite. Nitrite/nitrate (NO_2_^–^/NO_3_^–^) ratio was determined in the same muscle homogenate used for total ROS quantification. Nitrite was spectrophotometrically measured as a colored azo dye product at 540 nm (Anthos Zenyth 200, Biochrom, Cambridge, UK). This assay presents a 0 to 100 μM detection range, and a respective detection sensitivity of 0.222 μM and 0.625 μM for NO_2_^–^ and NO_3_^–^.

### Advanced Oxidation Protein Products, Protein, and Lipid Oxidative Damage Assay

Advanced oxidation protein products (AOPP) were analyzed as indicator of ROS-induced oxidative stress using a biochemical kit and the manufacturer’s instructions (OxiSelect AOPP Assay, Cell Biolabs, CA, USA). Briefly, 75 μL gastrocnemius homogenate was treated with 10 μL concentrated acetic acid and 5 μL KI at 1.16 M. All reactions were blocked after 5 min, and absorbances were read by spectrophotometry at 340 nm. A chloramine T-based standard curve ranging from 0 to 100 μM was obtained. The results were expressed as chloramine T equivalents.^33^

Protein oxidation was analyzed from protein carbonyl (PCN) quantification using a 2,4-dinitrophenylhydrazine (DNPH)-based colorimetric kit and its standardized protocol (Cayman Chemicals, MI, USA).^34^ In this assay, muscle pellets obtained after centrifuging the gastrocnemius homogenates were incubated for 15 min with 10 mM DNPH solution prepared in 0.5 M phosphoric acid (H_3_PO_4_). The reactions were analyzed by spectrophotometry at 385 nm in a 96-well microplate reader (Varioskan, Thermo Fisher Scientific, Waltham, Massachusetts, USA).

Lipid hydroperoxides (LPO) were measured as indicators of lipid oxidation in the precipitate (pellets) of gastrocnemius homogenate using a colorimetric LPO kit and the manufacturer’s instructions (Cayman Chemicals, MI, USA). Lipid hydroperoxides were extracted from muscle pellets using a solution containing 0.2 M HCl and 3% NH_4_SCN prepared in methanol, and 4.5 mM FeSO_4_ prepared in chloroform (1:1 v/v). All reactions were blocked after 5 min and read in a spectrophotometer at 500 nm. The results were obtained from a 13-hydroperoxy octadecadienoic acid-based standard curve ranging from 0 to 500 μM.^33^

### Histological Processing and Skeletal Muscle Histopathology

Gastrocnemius samples were immersed for 24 h in fresh histological fixative (4% paraformaldehyde prepared in 0.1 M Na_3_PO_4_ buffer, pH 7.2).^35^ Then, muscle fragments were included in glycol methacrylate. Longitudinal (vertical) muscle sections were obtained in a rotary microtome coupled with glass knives (Leica Biosystems, Wetzlar, Germany). Six semiserial sections were obtained for each animal, collecting one in every 30 sections to ensure the analysis of distinct histological areas. The sections were stained with hematoxylin and eosin (H&E) at 60 °C.^9,22^ The histopathological analysis was based on the microscopic observation (Axioscope A1, Carl Zeiss, Germany) of all histological slices by a pathologist using ×20 (×200 magnification) and ×40 (×400 magnification) objective lens. The histopathological findings were qualitatively reported considering: (i) parenchyma (skeletal myocytes) and stroma (connective tissue) distribution, (ii) inflammatory infiltrate, (iii) myocytes hypertrophy or hypotrophy, (iv) myonecrosis, and (v) presence and distribution of *T. cruzi* amastigote nests.^36,37^

### Muscle Histomorphometry and Second Order Stereology

The average thickness of skeletal myocytes was measured by computational planimetry, using the linear measurement tool of the Image-Pro Plus software (Media Cybernetics Inc., Silver Spring, MD, USA).^9,22^ Myocyte thickness (diameter—*D*) was evaluated in the central portion of 50 cells randomly sampled for each animal. The mean cross-sectional area (CSA) of skeletal myocytes was calculated as previously reported,^38^ using the formula CSA = π*r ^2^, where *r* is *D*/2. The volume density (*V*_v_, %) of muscle parenchyma and stroma was quantified from the stereological method, using the formula *V*_v_, % = Σ*P*_P_/*P*_T_, where Σ*P*_P_ is the number of points hitting the structure of interest (parenchyma or stroma) and *P*_T_ is the number of points used in the test system.^22,39^ A quadratic system containing 72 equidistant test points distributed in a 73 × 10^3^ μm^2^ test area was used.^26^ Twenty-four randomly sampled histological images (four in each histological section) were obtained for each animal at ×400 magnification using a bright field microscope (Axioscope A1, Carl Zeiss, Germany). The intensity of the muscular inflammatory infiltrate was estimated by quantifying the number of interstitial cell nuclei (ICN) as previously described.^39^ This parameter was measured in H&E-stained images using the stereological method according to the following formula: ICN = Σ_ICN_/*T*_A_, where Σ_ICN_ represent the number of interstitial cell nuclei counted in the test area (*T*_A_) used (here 25 × 10^3^ μm^2^).^22^ All analyses were conducted by a pathologist blinded to the intervention groups using the Image-Pro Plus software (Media Cybernetics Inc., Silver Spring, MD, USA).^26,40^

### Myeloperoxidase and *N*-Acetyl-β-d-glucosaminidase Muscular Activity Assay

Neutrophil and macrophage influxes were, respectively, estimated from myeloperoxidase (MPO) and *N*-acetyl-β-d-glucosaminidase (NAG) activity in gastrocnemius samples. NAG and MPO activities were measured using a biochemical colorimetric method, as previously detailed.^9^ NAG activity was measured in muscle samples, which were frozen in liquid nitrogen, pulverized in a crucible, and homogenized in Na_3_PO_4_ buffer containing protease inhibitor (Sigma-Aldrich, St. Louis, MO, USA). The homogenate was centrifuged for 15 min at 3000*g* and 4 °C. NAG activity in the supernatant was measured using a 96-well biochemical kit and the manufacturer’s instructions (Abcam, Cambridge, UK). This assay uses a synthetic *p*-nitrophenol derivative (R-*p*NP) as NAG substrate, generating a reaction product (pNP) spectrophotometrically detected at 400 nm.^9^

In the MPO assay, muscle pellets obtained in the NAG assay were weighed and homogenized in the reaction buffer (0.1 M NaCl, 0.015 M Na_2_-EDTA, and 0.02 M Na_3_PO_4_, at pH 4.7). The homogenate was centrifuged at 12,000*g* and 4 °C for 10 min. The supernatant was discarded, and the pellets were resuspended in 0.5% hexa-1,6-*bis*-decyltrimethylammonium bromide prepared in 0.05 M Na_3_PO_4_ buffer. The mixture was reacted with 0.3 mM H_2_O_2_ dissolved in pH 6.0 Na_3_PO_4_ buffer and 1.6 mM 3,3′-5,5′-tetramethylbenzidine prepared in DMSO. MPO activity was measured by spectrophotometry at 450 nm using a 96-well microplate reader (Varioskan, Thermo Fisher Scientific, Waltham, Massachusetts, USA).

### Cytokines Immunoassay

Gastrocnemius samples were homogenized in the presence of protease inhibitor (Sigma-Aldrich, St. Louis, MO, USA) and centrifuged at 3000*g* for 15 min and 4 °C. The cytokines interleukin 6 (IL-6), interleukin-10 (IL-10), interferon gamma (IFN-γ), and tumor necrosis factor (TNF) were quantified in muscle homogenate. These molecules were analyzed by cytometric bead array (CBA) using a mouse Th1/Th2/Th17 assay and the manufacturer’s instructions (BD Biosciences, San Diego, CA, USA). The data were collected in the FACSVerse flow cytometer (Biosciences, San Diego, CA, USA) and analyzed in the FCAP 3.0 software. Standard curves were obtained using recombinant cytokines ranging from 20 to 5000 pg/mL.^22^

### Anti-*T. cruzi* Immunoglobulin Assay

Anti-*T. cruzi* immunoglobulin G (IgG) was quantified in blood serum by enzyme-linked immunosorbent assay (ELISA) (Bethyl Laboratories, Montgomery, Texas, USA) as previously reported.^37^ Briefly, 96-well polystyrene microplates were coated with 3 μg of *T. cruzi* antigens obtained by alkaline extraction in blood trypomastigotes isolated from previously infected mice in the peak of parasitemia.^41^ *T. cruzi* antigens were incubated for 12 h with 5 μL of blood serum from each animal. Peroxidase-conjugated antimouse IgG detection antibodies (Bethyl Laboratories, Montgomery, Texas, USA) were added to each well and incubated at 37 °C for 4 h. Finally, the chromogenic substrate *o*-phenylenediamine dihydrochloride was added, and the reaction was read by spectrophotometry at 490 nm (Varioskan, Thermo Fisher Scientific, Waltham, Massachusetts, USA). Serum samples collected from uninfected mice were used as negative controls. Anti-IgG antibodies were omitted from control reactions, and absorbances were subtracted from the final results.^37^

### Statistical Method

Mean and standard deviation of the mean (mean ± S.D.), and median and interquartile range were used to express all results. Data distribution was characterized using the D’Agostino–Pearson method. Data variance was analyzed using the one-way analysis of variance (One-way ANOVA) technique followed by the Student–Newman–Keuls posthoc method. Multiple comparisons from nonparametric data were conducted using the Kruskal–Wallis *H*-test. The results of all tests presenting a *P* value ≤0.05 were admitted as statistically significant.

## Results

As shown in Figure 4, physicochemical modeling indicated that ME presented characteristics compatible with the oral bioavailability of drugs, since it respected the optimal range that indicates adequate conditions of lipophilicity, size, polarity, solubility, flexibility, and saturation for this type of drug. Although Bz fell within the optimal ranges for most of these parameters, the limits for saturation were exceeded. In addition, pharmacodynamic and pharmacokinetic prediction revealed important similarities between ME and Bz (Table 1). These molecules exhibited limited permeability in target mammalian cells (Caco-2 and MDCK), acceptable passive permeability (considering TPSA and log­(*P*)), and good potential for intestinal absorption, bioavailability, potential permeability across the blood–brain barrier (BBB), noninteraction with P-glycoprotein, and drug half-life. Only ME presented an optimal pH-independent lipophilicity, exhibited higher clearance, as well as greater potential interaction and transport via plasma proteins compared with Bz. Furthermore, acceptors and donors distribution, as well as the molecular mass, log­(*P*), and TPSA for ME and BZ were consistent with Lipinski’s rule of five for orally active drugs.

**Figure 4:**
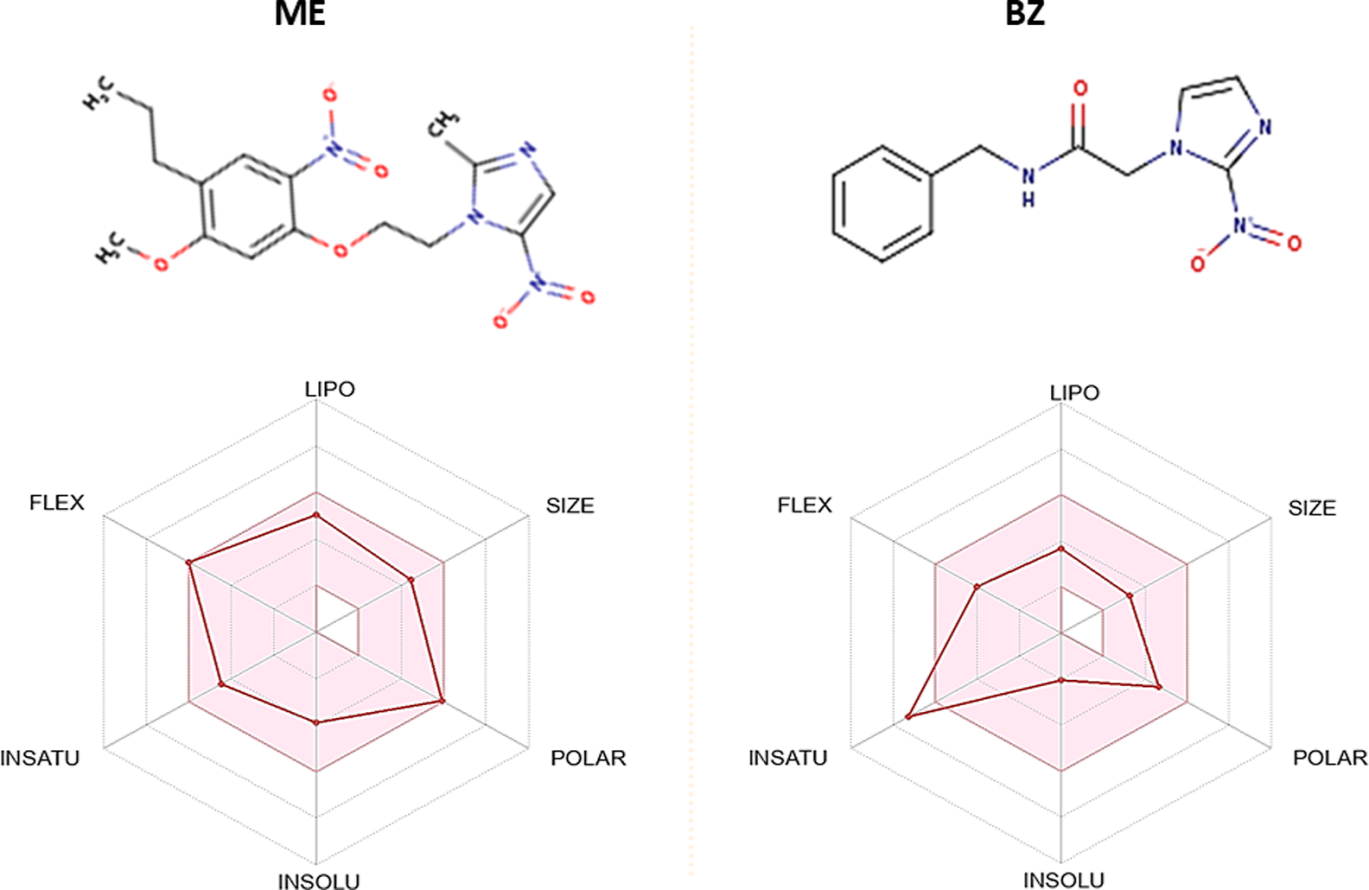
Predictive physicochemical characteristics of the novel nitroimidazole-based drug 1-(2-(2-methoxy-6-nitro-4-propylphenoxy)­ethyl)-2-methyl-5-nitro-1*H*-imidazole (ME) compared to the reference trypanocidal drug benznidazole (BZ). The pink zone in the bioavailability radar is the suitable physicochemical space for oral bioavailability based on the optimal range for lipophilicity, size, polarity, insolubility, instauration, and flexibility.

**Table 1:** Predictive Pharmacodynamic and Pharmacokinetic Characteristics of the Novel Nitroimidazole-Based Drug 1-(2-(2-Methoxy-6-nitro-4-propylphenoxy)­ethyl)-2-methyl-5-nitro-1*H*-imidazole (ME) Compared to the Reference Trypanocidal Drug Benznidazole (BZ)^a^ ^,^ ^b^ ^,^ ^c^

parameter*	ME	BZ
Caco-2 (log *P*_app_)	–4.41	–4.47
MDCK cell permeability (cm/s)	–3.24	–3.27
intestinal absorption (%)	absorbed	absorbed
oral bioavailability	bioavailable	bioavailable
plasma protein binding	64.41	51.31
blood–brain barrier	penetrable	penetrable
P-glycoprotein inhibitor	noninhibitor	noninhibitor
P-glycoprotein subtract	nonsubstrate	nonsubstrate
log(*D*) at pH = 7.4 (log mol/L)	3.16	1.25
log(*P*) (log mol/L)	2.82	0.76
log(*S*) (log mol/L)	–3.64	–2.57
p*K*_a_ acid (−log *K*_a_)	7.43	8.47
p*K*_a_ basic (−log *K*_a_)	3.76	4.35
clearance	6.17	2.28
organic cation transporter 2	noninhibitor	inhibitor
half-life of drug	<3 h	<3 h
molecular weight	364.358	260,253
TPSA	122.56	90.06
hydrogen bond donors (n)	0	1
hydrogen bond acceptors (n)	10	7

a TPSA: topological polar surface area.

b ME SMILES: Cc1ncc­([N+]­(=O)­[O–])­n1CCOc2 cm^3^(OC)­c­(cc2­[N+]­(=O)­[O–])­CCC.

c BZ SMILES: C1=CC=C­(C=C1)­CNC­(=O)­CN2C=CN=C2­[N+]­(=O)­[O-].

The antiparasitic assay revealed a 7.70 μM IC_50%_ for ME and 5.15 μM IC_50%_ for Bz against blood trypomastigotes in vitro. As shown in Figure 5, our in vitro results also indicated a high rate of *T. cruzi* viability, infectivity, and parasite load in host cells exposed to untreated parasites. Conversely, these parameters were markedly attenuated by Bz and ME treatment (*P* < 0.05), which exhibited a dose-dependent effect. As expected for a reference drug, Bz induced the most prominent antiparasitic effects. However, the highest ME concentration determined similar antiparasitic results from a dose approximately 29% lower than that of Bz.

**Figure 5:**
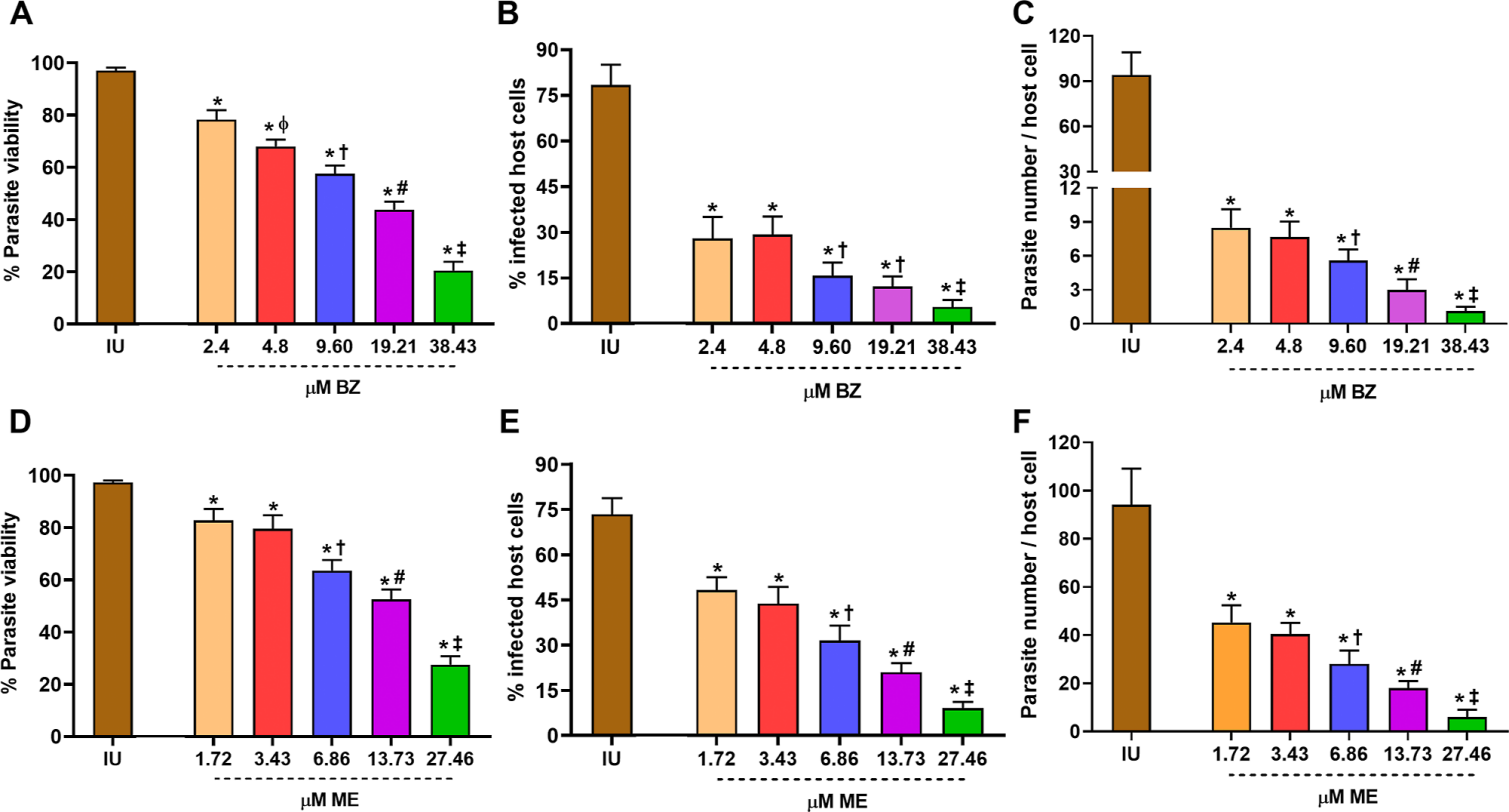
Effect of the novel nitroimidazole-based drug 1-(2-(2-methoxy-6-nitro-4-propylphenoxy)­ethyl)-2-methyl-5-nitro-1*H*-imidazole (ME) and benznidazole (BZ) treatment on parasite viability (A,D), host cells infection rate (B,E), and cell parasitism (C,F) in trypomastigotes and C2C12 skeletal myocytes in vitro. Data are expressed as mean and standard deviation. Statistical difference among the groups (*P* < 0.05), compared to * infected untreated (IU), φ 2.4 μM, † 2.4 and 4.8 μM, # 2.4, 4.8, and 9.6 μM, ‡ 2.4, 4.8, 9.6, and 19.21 μM in BZ-treated parasites/cells; and compared to * IU, † 1.72, and 3.43 μM, # 1.72, 3.43, and 6.86 μM, and ‡ 1.72, 3.43, 6.86, and 13.73 μM in ME-treated parasites/cells.

In vivo, all *T. cruzi*-infected and untreated animals showed high parasitemia and skeletal muscle parasite load (Figure 6). Treatments with ME and mainly with Bz administered alone were efficient in reducing parasitemia and muscle parasitism (*P* < 0.05). However, the most prominent effects were achieved by combining these drugs, especially using the highest ME dose (100 mpk) combined with the standard Bz dose (100 mpk), which significantly reduced blood and muscle parasitism compared to the other groups (*P* < 0.05).

**Figure 6:**
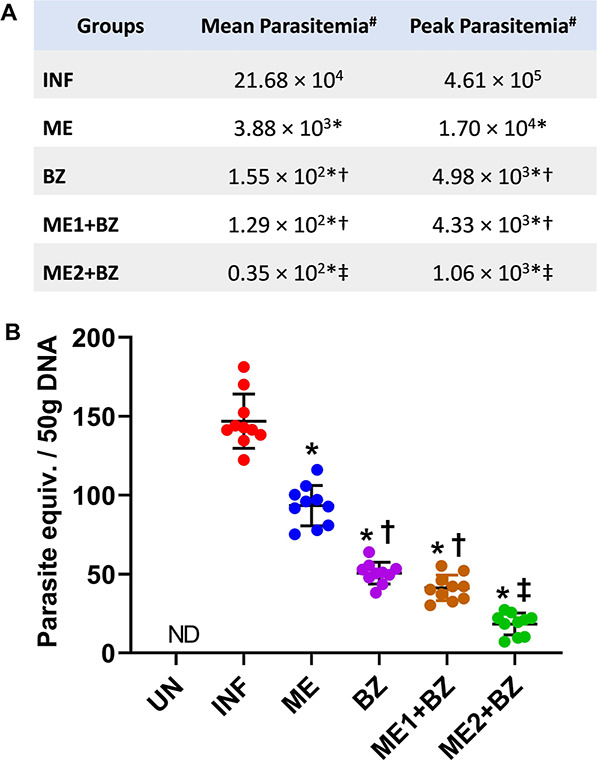
Blood (A) and skeletal muscle (B) parasitism in *T. cruzi*-infected mice untreated and treated with the novel nitroimidazole-based drug 1-(2-(2-methoxy-6-nitro-4-propylphenoxy)­ethyl)-2-methyl-5-nitro-1*H*-imidazole (ME) alone and combined with benznidazole (BZ). Groups: UN = uninfected, INF = infected untreated, ME = infected treated with 100 mpk ME, BZ = infected treated with 100 mpk Bz, ME1 + BZ = infected treated with 50 mpk ME + 100 mpk Bz, and ME2 + BZ = infected treated with 100 mpk ME + 100 mpk Bz. Data are expressed as median (A) and mean and standard deviation (B, each point indicates individual values). Statistical difference among the groups (*P* < 0.05), compared to * INF, † ME, ‡ ME, BZ, and ME1 + BZ.

Histopathological analysis (Figure 7) indicated that skeletal muscle from uninfected and untreated mice exhibited a normal microstructure, characterized by well-defined myocytes with homogeneous eosinophilic cytoplasm, evident transverse striation, scant connective stroma, and limited interstitial cellularity (Figure 7). In contrast, untreated infected animals presented prominent skeletal myositis, determined by multiple intracellular *T. cruzi* nests, intense diffuse inflammatory infiltrate, formed predominantly by mononuclear cells, expansion of the connective stroma, skeletal myocyte hypotrophy, and retraction (cytoplasmic irregularities/undulations). All of these changes were markedly attenuated by treatment with Bz and ME administered alone or in combination. Monotherapy or drug combination using the lowest ME dose (ME1 + Bz) prominently reduced skeletal myositis, determining a limited presence of inflammatory foci. However, the highest ME dose combined with Bz (ME2 + Bz) exerted the most potent myoprotective effect, characterized by a skeletal muscle microstructure similar to that observed in uninfected control animals (Figure 7).

**Figure 7:**
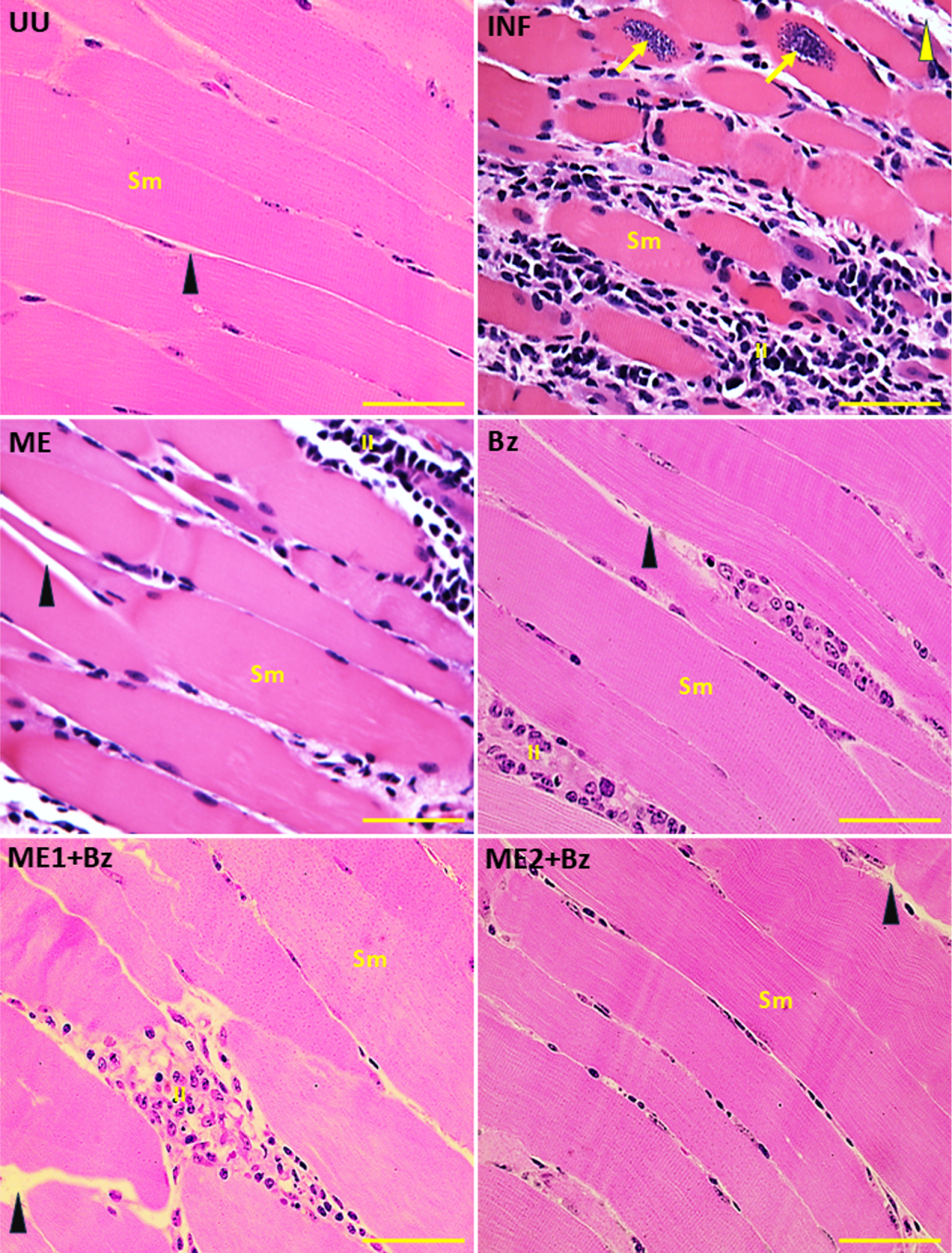
Microscopic images of the skeletal muscle from uninfected and *T. cruzi*-infected mice, untreated and treated with the novel nitroimidazole-based drug 1-(2-(2-methoxy-6-nitro-4-propylphenoxy)­ethyl)-2-methyl-5-nitro-1*H*-imidazole (ME) alone and combined with benznidazole (BZ) (hematoxylin and eosin staining, bright field microscopy, scale bars = 50 μm). Groups: UN = uninfected, INF = infected untreated, ME = infected treated with 100 mpk ME, BZ = infected treated with 100 mpk Bz, ME1 + BZ = infected treated with 50 mpk ME + 100 mpk Bz, and ME2 + BZ = infected treated with 100 mpk ME + 100 mpk Bz. Arrow = *T. cruzi* nest. Arrowhead = interstitial space. Sm= skeletal myocyte. II = inflammatory infiltrate.

Quantitative microstructural analysis (Figure 8) corroborated the histopathological evidence, revealing a higher density of interstitial/inflammatory cells, a reduction in the area occupied by muscle parenchyma, and in the thickness and cross-sectional area of myocytes in the skeletal muscle of the infected animals compared to control uninfected animals (*P* < 0.05). All of these parameters were mitigated by the treatments administered (*P* < 0.05). Animals treated with Bz alone or combined with the lowest ME dose (ME1 + Bz) showed reduced interstitial cellularity/inflammatory cells and parenchymal loss compared to the ME group (*P* < 0.05). This parameter was even more reduced in ME2 + Bz animals compared to the other treatment groups (*P* < 0.05). Myocytes thickness and cross-sectional area were similar in control uninfected animals and all treatment groups (*P* > 0.05).

**Figure 8:**
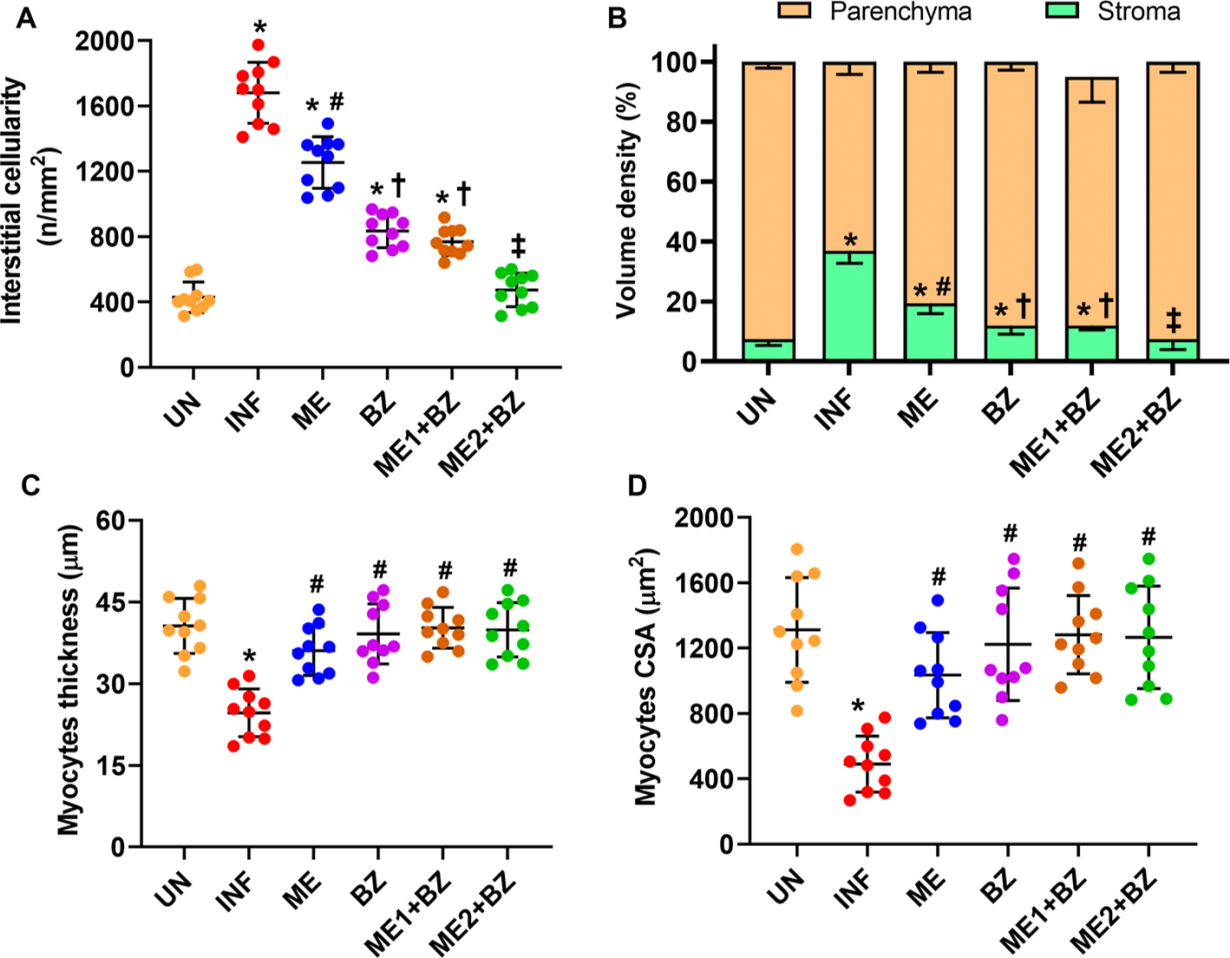
Interstitial cellularity (A), parenchyma and stroma distribution (B), myocytes thickness (C), and cross-sectional area (CSA—D) in the skeletal muscle from *T. cruzi*-infected mice untreated and treated with the novel nitroimidazole-based drug 1-(2-(2-methoxy-6-nitro-4-propylphenoxy)­ethyl)-2-methyl-5-nitro-1*H*-imidazole (ME) alone and combined with benznidazole (BZ). Groups: UN = uninfected, INF = infected untreated, ME = infected treated with 100 mpk ME, BZ = infected treated with 100 mpk Bz, ME1 + BZ = infected treated with 50 mpk ME + 100 mpk Bz, and ME2 + BZ = infected treated with 100 mpk ME + 100 mpk Bz. Data are expressed as mean and standard deviation (each point indicates individual values). Statistical difference among the groups (*P* < 0.05), compared to * UN, # INF, † ME, ‡ ME, BZ, and ME1 + BZ.

As shown in Figure 9, uninfected animals presented reduced ROS and NO levels, which were markedly increased in infected untreated mice compared to all groups (*P* < 0.05). Alone, Bz was more effective than ME in reducing these parameters (*P* < 0.05). However, ROS levels were significantly attenuated in ME2 + Bz mice compared to the other groups (*P* < 0.05). In addition, treatments with Bz alone or combined with ME were equally effective in reducing NO levels compared to animals receiving ME alone (*P* < 0.05).

**Figure 9:**
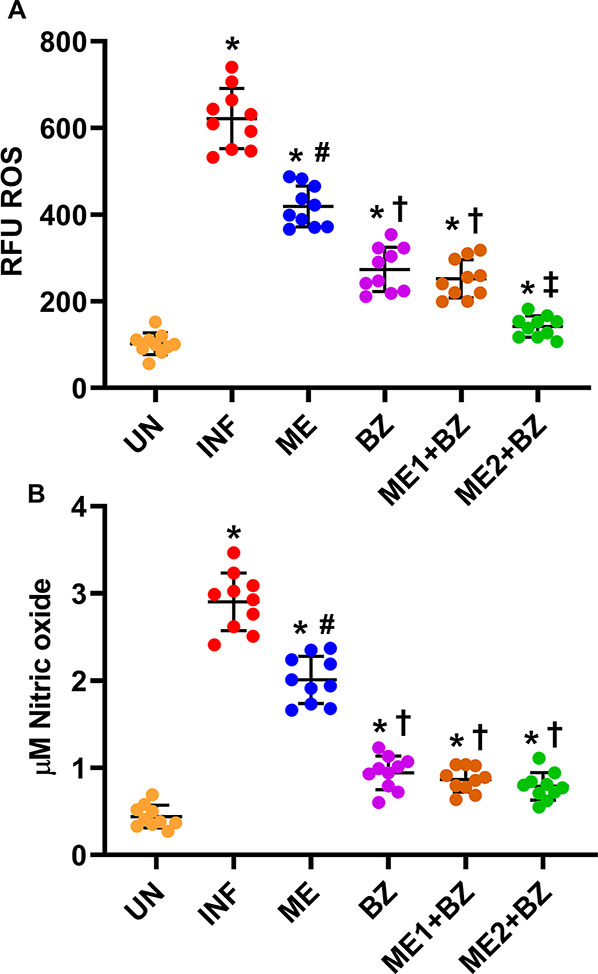
Reactive oxygen species (ROS—A) and nitric oxide (B) levels in the skeletal muscle from *T. cruzi*-infected mice untreated and treated with the novel nitroimidazole-based drug 1-(2-(2-methoxy-6-nitro-4-propylphenoxy)­ethyl)-2-methyl-5-nitro-1*H*-imidazole (ME) alone and combined with benznidazole (BZ). Groups: UN = uninfected, INF = infected untreated, ME = infected treated with 100 mpk ME, BZ = infected treated with 100 mpk Bz, ME1 + BZ = infected treated with 50 mpk ME + 100 mpk Bz, and ME2 + BZ = infected treated with 100 mpk ME + 100 mpk Bz. RFU = relative fluorescence units. Data are expressed as mean and standard deviation (each point indicates individual values). Statistical difference among the groups (*P* < 0.05), compared to * UN, # INF, † ME, ‡ ME, BZ, and ME1 + BZ.

Biochemical analysis indicated intense oxidation of proteins (AOPP and PCN levels) and lipids (lipid hydroperoxide levels) in the skeletal muscle of untreated *T. cruzi*-infected animals compared to the other groups (*P* < 0.05) (Figure 10). These parameters were significantly attenuated in all treated groups (*P* < 0.05). Animals treated with Bz alone or combined with the lowest ME dose (ME1 + Bz) showed lower AOPP, PCN, and lipid hydroperoxide levels compared to the ME group (*P* < 0.05). However, AOPP and lipid showed even lower levels in the ME2 + Bz group compared to the other treated groups (*P* < 0.05).

**Figure 10:**
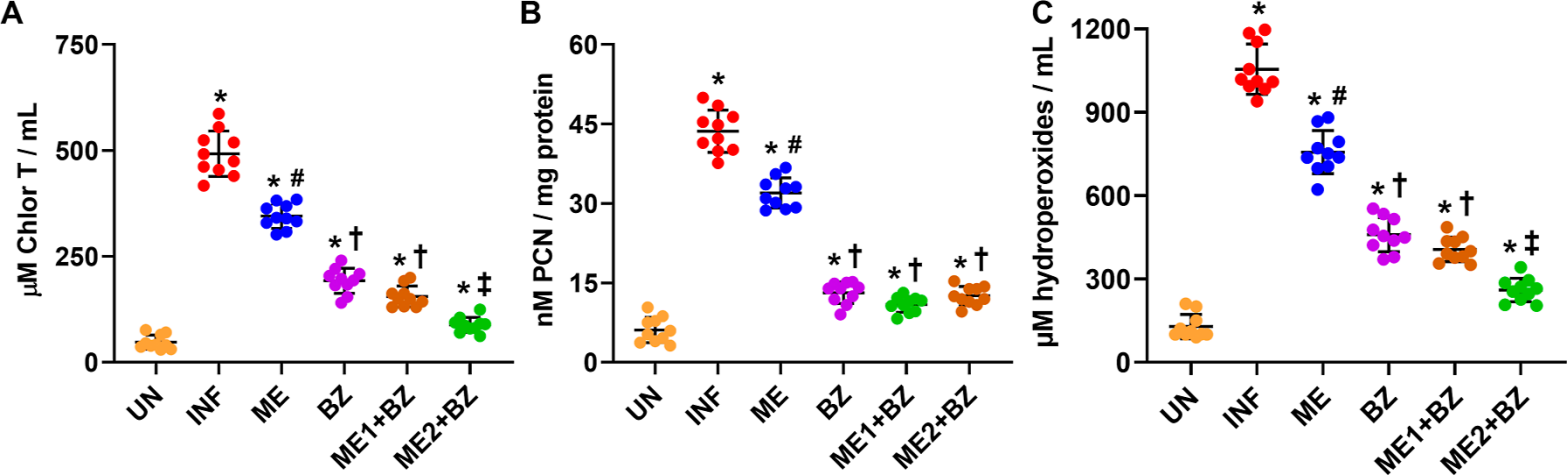
Advanced oxidation protein products (chloramine T equivalents—Chlor T, A), protein carbonyl (PCN, B), and lipid hydroperoxides in the skeletal muscle from *T. cruzi*-infected mice untreated and treated with the novel nitroimidazole-based drug 1-(2-(2-methoxy-6-nitro-4-propylphenoxy)­ethyl)-2-methyl-5-nitro-1*H*-imidazole (ME) alone and combined with benznidazole (BZ). Groups: UN = uninfected, INF = infected untreated, ME = infected treated with 100 mpk ME, BZ = infected treated with 100 mpk Bz, ME1 + BZ = infected treated with 50 mpk ME + 100 mpk Bz, and ME2 + BZ = infected treated with 100 mpk ME + 100 mpk Bz. Data are expressed as mean and standard deviation (each point indicates individual values). Statistical difference among the groups (*P* < 0.05), compared to * UN, # INF, † ME, ‡ ME, BZ, and ME1 + BZ.

As indicated in Figure 11, MPO and NAG activity was increased in infected untreated animals compared to the other groups (*P* < 0.05). These parameters were markedly attenuated in all treated groups (*P* < 0.05). MPO activity was similarly reduced in animals receiving Bz alone or combined with both ME doses compared to the group treated with ME alone (*P* < 0.05). MPO activity was reduced in the group ME2 + Bz compared to the other treatment groups (*P* < 0.05).

**Figure 11:**
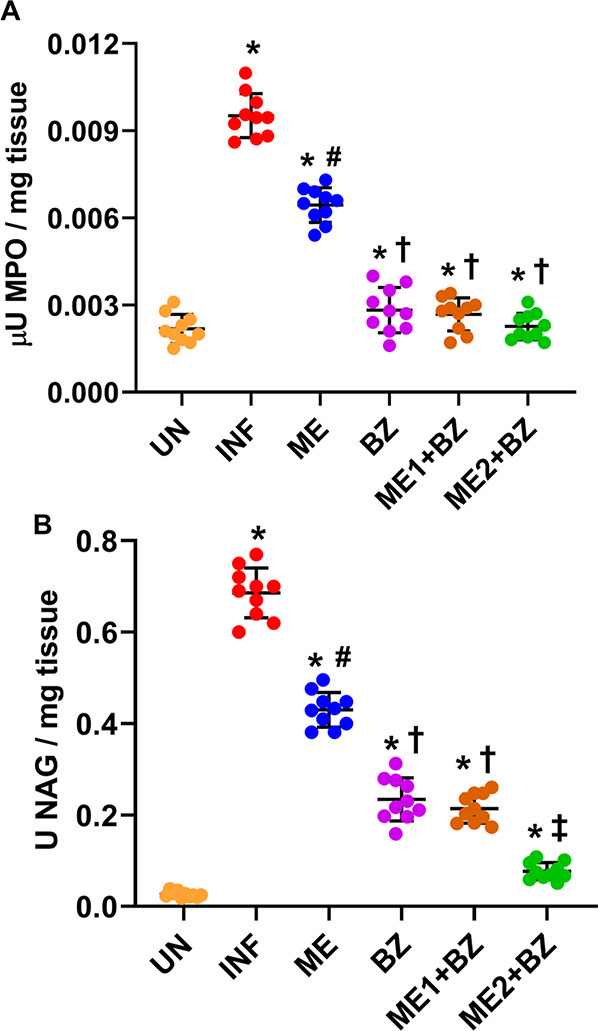
Myeloperoxidase (MPO—A) and *N*-acetyl-β-d-glucosaminidase (NAG, B) activity in the skeletal muscle from *T. cruzi*-infected mice untreated and treated with the novel nitroimidazole-based drug 1-(2-(2-methoxy-6-nitro-4-propylphenoxy)­ethyl)-2-methyl-5-nitro-1*H*-imidazole (ME) alone and combined with benznidazole (BZ). Groups: UN = uninfected, INF = infected untreated, ME = infected treated with 100 mpk ME, BZ = infected treated with 100 mpk Bz, ME1 + BZ = infected treated with 50 mpk ME + 100 mpk Bz, and ME2 + BZ = infected treated with 100 mpk ME + 100 mpk Bz. Data are expressed as mean and standard deviation (each point indicates individual values). Statistical difference among the groups (*P* < 0.05), compared to * UN, # INF, † ME, ‡ ME, BZ, and ME1 + BZ.

Cytokine analysis (Figure 12) revealed elevated levels of IFN-γ, TNF, IL-6, and IL-10 in the skeletal muscle of untreated infected animals compared to the other groups (*P* < 0.05). All of these cytokines were down-regulated by monotherapy or combination therapy with Bz and ME (*P* < 0.05). Animals treated with Bz alone or combined with the lowest ME dose (ME1 + Bz) presented reduced IFN-γ, TNF, and IL-10 levels compared to the ME group (*P* < 0.05). This parameter was even more reduced in ME2 + Bz animals compared to the other treatment groups (*P* < 0.05). IL-6 levels were similarly attenuated in ME1 + Bz and ME2 + Bz animals compared to the other treatment groups (*P* < 0.05).

**Figure 12:**
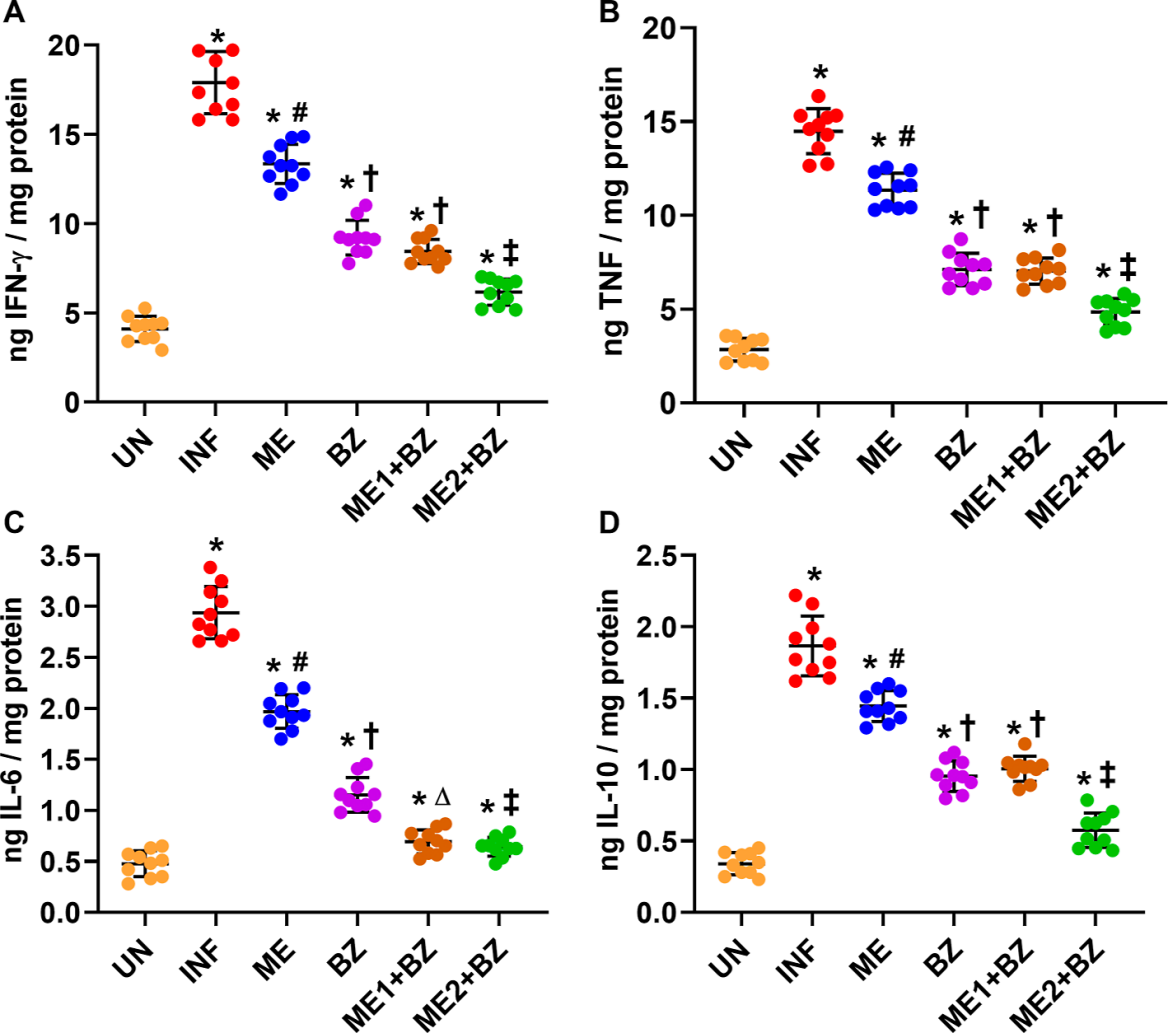
Interferon gamma (IFN-γ—A), tumor necrosis factor (TNF—B), interleukin 6 (IL-6—C), and interleukin 10 (IL-10—D) levels in the skeletal muscle from *T. cruzi*-infected mice, untreated, and treated with the novel nitroimidazole-based drug 1-(2-(2-methoxy-6-nitro-4-propylphenoxy)­ethyl)-2-methyl-5-nitro-1*H*-imidazole (ME) alone and combined with benznidazole (BZ). Groups: UN = uninfected, INF = infected untreated, ME = infected treated with 100 mpk ME, BZ = infected treated with 100 mpk Bz, ME1 + BZ = infected treated with 50 mpk ME + 100 mpk Bz, and ME2 + BZ = infected treated with 100 mpk ME + 100 mpk Bz. Data are expressed as mean and standard deviation (each point indicates individual values). Statistical difference among the groups (*P* < 0.05), compared to * UN, # INF, † ME, ‡ ME, BZ, and ME1 + BZ.

Measurements of renal function markers (Figure 13) indicated similar ALT and AST plasma levels in uninfected control animals, untreated infected animals, and those treated with ME alone. The levels of these enzymes were higher in the groups treated with Bz alone or combined with the lowest ME dose (ME1 + Bz) (*P* < 0.05). These levels were even more pronounced in the group ME2 + Bz (*P* < 0.05). Creatine and urea serum levels were similar in all groups (*P* > 0.05).

**Figure 13:**
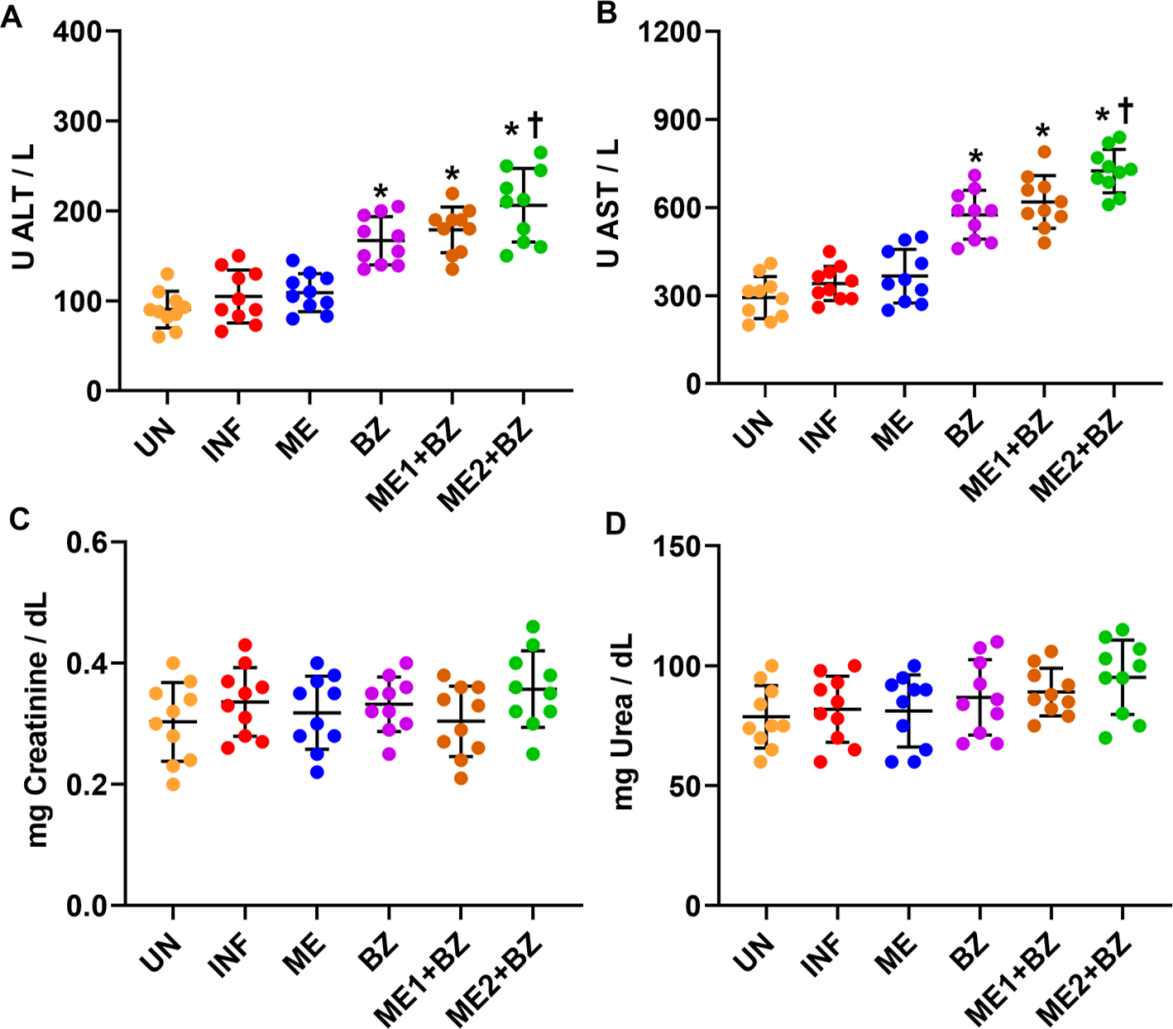
Biochemical markers of drug toxicity in *T. cruzi*-infected mice, untreated and treated with the novel nitroimidazole-based drug 1-(2-(2-methoxy-6-nitro-4-propylphenoxy)­ethyl)-2-methyl-5-nitro-1*H*-imidazole (ME) alone and combined with benznidazole (BZ). Groups: UN = uninfected, INF = infected untreated, ME = infected treated with 100 mpk ME, BZ = infected treated with 100 mpk Bz, ME1 + BZ = infected treated with 50 mpk ME + 100 mpk Bz, and ME2 + BZ = infected treated with 100 mpk ME + 100 mpk Bz. Data are expressed as mean and standard deviation (each point indicates individual values). Statistical difference among the groups (*P* < 0.05), compared to * UN, INF, ME, and † BZ.

## Discussion

Using a systematic molecular hybridization method, we developed an innovative nitroimidazole derivative (ME) that demonstrated a potent antiparasitic effect in vitro and in vivo. As ME respected all physicochemical limits analyzed, our findings reinforce that the chemical synthesis strategy was efficient in producing a novel nitroimidazole-based drug with characteristics potentially favorable for oral administration. In addition, ME also exhibited acceptable potential for passive permeability, absorption, bioavailability, and half-life, which were similar to those of Bz. ME absorption parameters were improved by its nonsubstrate nature, reinforcing its potential for intracellular activity considering a lower elimination risk by P-glycoprotein, an ATP-dependent transmembrane efflux pump.^42^ ME also presented potential for BBB penetration, which is a relevant characteristic considering that cells of the central nervous system can be infected and often establish intracellular *T. cruzi* reservoirs.^43^ Thus, more effective antiparasitic drugs need to cross the BBB to access these cells and eliminate the parasite, preventing infection recurrence years or decades after initial contact with *T. cruzi*.^43,44^ Despite its greater potential for systemic distribution bound to plasma proteins, the ME clearance was higher than that of Bz. This characteristic may be linked to weak molecular interactions, favorable water solubility (evidenced by log *D*, log *P*, and log *S*), and renal excretion due to the noninhibitory potential on organic cation transporter 2,^42^ unlike the inhibitory interaction predicted for BZ. This proposition is also reinforced by the half-life similarity between ME and Bz.

The direct antiparasitic properties of this drug were proven in vitro by attenuating *T. cruzi* viability and its ability to infect skeletal muscle cells. Although the antiparasitic effect of Bz was prominent, ME treatment achieved exceptional results from doses approximately 29% lower than those of Bz, in addition to demonstrating a desirable dose-dependent behavior similar to that of Bz. Furthermore, this drug was effective in limiting the intracellular parasite load, reinforcing its potential to compromise the replicative capacity of these parasites within the host cells. These findings reinforce ME cytotoxicity against trypomastigotes and amastigotes, the two parasitic forms with the greatest clinical relevance in the infection of vertebrate hosts.^45^ Although desirable, this is not a trivial characteristic, since different evolutionary forms of *T. cruzi* exhibit marked variability in drug resistance and frequently show divergent responses to antiparasitic chemotherapy.^1,5^ Accordingly, toxicity against trypomastigotes and amastigotes is essential for the development of antiparasitic compounds targeted at ChD treatment, supporting their use in monotherapy and/or combination chemotherapy.^46,47^

In accordance with our in vitro findings, ME treatment also achieved a potent antiparasitic effect in vivo. Although Bz alone demonstrated greater efficacy in attenuating parasitemia and parasite load compared to ME monotherapy, the combination of Bz with the higher ME dose determined a superior antiparasitic response. As with parasitemia, the attenuation of muscle parasite load represents an important response to antiparasitic chemotherapy.^1,39^ It is well established that complete parasitemia suppression is not an unequivocal marker of parasitological cure, particularly during the chronic phase of Chagas disease.^48^ Accordingly, *T. cruzi* reservoirs frequently establish themselves in multiple organs, including skeletal muscles, even in the absence of patent parasitemia.^6,45^ Thus, *T. cruzi* infection chronicity has been associated with the persistence of nests formed by chemotherapy-resistant parasites.^49^ These nests are frequently constituted by dormant parasites, which are capable of reactivating their replicative process and re-establishing parasitemia within a variable period after the end of treatment,^49,50^ a response potentially conditioned by the permissiveness of the host’s immune response.^49,51^ In this context, the effectiveness of the Bz and ME combination in reducing the parasite load and inhibiting the formation of *T. cruzi* reservoirs/nests in the parasitized organs was consistent with the desired responses of trypanocidal drugs capable of preventing the chronicity of the infection and achieving a cure for ChD.^11,52,53^ Although further mechanistic studies are needed, our findings align with the rationale supporting combination chemotherapy, which posits that complementary, additive, or synergistic pharmacological properties of chemically distinct drugs are centrally linked to antiparasitic effects superior to monotherapy.^5,11,14^

The efficacy of the investigated drugs was also evidenced in morphological analysis, which revealed a combination of antiparasitic, anti-inflammatory, and myoprotective effects. Accordingly, treatment with Bz, ME, and especially the combined use of these drugs attenuated or prevented the formation of amastigote nests, myositis, as well as disorganization, hypotrophy, and loss of skeletal myocytes. Thus, the muscle histoarchitecture was completely preserved when combining Bz with the highest ME dose, maintaining a microstructural pattern similar to that of the uninfected control group. Tissue microstructure preservation is a central element in mitigating the deterioration of muscle function identified in ChD.^10,40,54^ There is evidence that muscle parasitism causes extensive damage to the muscle parenchyma, in which myocyte loss is associated with myonecrosis and myocytolysis induced by direct parasitism of infected cells, in addition to indirect destructive events mediated by nonspecific oxy-inflammatory defense reactions against *T. cruzi*.^8,54^ As typically observed for Bz^14^ and other nitro compounds such as fexinidazole^55^ and nifurtimox,^56^ the ME-induced myoprotective effect appears to be linked to the combination of direct antiparasitic and anti-inflammatory effects, which seem to recover the fundamental properties of the drugs used in the construction of this new molecular hybrid.^14,25,31^ In this sense, there is evidence that drugs capable of reducing the parasite number in infected organs through direct cytotoxic effects are able to reduce the antigenic load, decreasing the intensity of the immune response that triggers oxy-inflammatory tissue damage.^57,58^ However, a possible direct anti-inflammatory effect induced by ME cannot be ruled out, which has already been proven for Bz in association with its classic antiparasitic effect.^14^

The levels of reactive mediators and markers of lipid and protein oxidation corroborate our hypothesis, indicating that treatment with Bz and ME alone and especially in combination determined a remarkable attenuation of muscle oxidative stress. These findings may partially explain the prominent myoprotective effect of this pharmacological combination, since in addition to impairing cellular function, lipid and protein oxidative damage are important determinants of myocyte death (myonecrosis) and connective tissue expansion/fibrosis in ChD.^39,54^ Oxidative and nitrosative stress have a complex and multifactorial origin in Chagas disease.^59^ There is evidence that the parasite is capable of inducing direct mitochondrial dysfunction in infected cells, leading to excessive production of reactive species due to instability in mitochondrial enzyme complexes.^54,59^ Furthermore, the immune cells themselves produce reactive species as an important line of defense against *T. cruzi*.^60^ However, these molecules are also cytotoxic to host cells, which also become a target of this nonspecific pro-oxidant response.^61,62^ Therefore, drugs or pharmacological strategies that combine antiparasitic, anti-inflammatory, and antioxidant effects may be relevant to attenuate tissue damage while controlling infection.^63^ Although Bz has a proven anti-inflammatory potential, paradoxically this drug also exhibits a potent pro-oxidant effect, which is linked to its processing by *T. cruzi* nitroreductases, leading to the formation of reactive metabolites (e.g., dialdehyde glyoxal) that are toxic to the parasite and host cells.^62^ Part of the toxicity associated with Bz administration for ChD treatment is linked to this mechanism, reinforcing the need to develop drugs with more favorable pharmacodynamic properties and reduced side effects.^11^

Quantification of inflammatory markers reinforces the proposition that the myoprotective effects induced by Bz and ME treatments were associated with control of the oxy-inflammatory response. In this sense, MPO and NA activity in skeletal muscle was prominently attenuated by the combination of these drugs at the highest ME dose. These findings are consistent with the remarkable control of muscle inflammatory infiltrate induced by combination chemotherapy, since high MPO and NAG levels are indicators of increased leukocyte recruitment and activation in *T. cruzi*-infected organs, specifically neutrophils and macrophages.^64^ Interestingly, these cells are directly involved in the production of reactive species (e.g., OH^•–^, O_2_^•–^, ONOO^•–^, H_2_O_2_, HClO, and NO) against *T. cruzi*,^61^ whose levels were consistently reduced following the inhibition of muscle inflammatory infiltrate. This response was also consistent with the muscle levels of all cytokines investigated, which were attenuated in all animals treated with Bz and ME, with predominant effects in the groups receiving these drugs in combination. Increased IFN-γ, TNF, and IL-6 levels corroborate the development of an immunological response pattern typically observed in ChD,^65^ which seeks to protect the host by stimulating the recruitment and activation of mononuclear and polymorphonuclear leukocytes, which are important for innate and acquired defense against *T. cruzi*.^66,67^ There is evidence that deficient production of these cytokines increases susceptibility to *T. cruzi* infection, especially by inhibiting the activation and *trans*-endothelial migration of lymphocytes to infected organs and the polarization of macrophages to the classic M1 phenotype.^68,69^ In addition, IFN-γ, TNF, and IL-6 deficiency impair NO production by these cells, which is essential in the protective response against *T. cruzi*.^70^

In the absence of treatment, low IFN-γ, TNF, and IL-6 levels are detrimental to the host, as they favor *T. cruzi* replication and dissemination, accelerating the progression and severity of infection by this pathogen.^71,72^ However, down-regulation of these cytokines is frequently associated with effective antiparasitic treatments. Accordingly, less activation of the immune response is expected with the reduction of the antigenic load determined by better pharmacological control of parasitemia and tissue parasitism.^65^ This response is also important for regulating the intensity of the tissue inflammatory response, minimizing the risk of immune hyperactivation, which frequently determines the extensive oxy-inflammatory cell damage identified in untreated *T. cruzi* infection.^73^ Given the effectiveness of the treatment in attenuating the levels of the main markers of the Th1 response (IFN-γ and TNF), a concomitant adjustment of IL-10 levels is natural and expected, reinforcing the proposition of a myoprotective effect that also involves the control of the skeletal muscle inflammatory response.^74,75^ IL-10 modulation is essential to regulate the relationship between susceptibility and resistance to *T. cruzi* infection.^71^ Accordingly, excessive levels of this cytokine inhibit pro-inflammatory defense responses against the parasite, while IL-10 depletion favors the development of severe oxy-inflammatory reactions and microstructural destructive processes that frequently increase host mortality rates.^76^ There is consistent evidence that the efficacy of Bz-based reference chemotherapy manifests as a response to the interaction between the drug-induced effects and the host’s immune defenses.^77^ Thus, ME administration showed similar behavior to that expected for Bz monotherapy, reinforcing the proposition that ME treatment (especially in combination with Bz) maintains the balance of the host’s immune response and its protective function as the infection is controlled by chemotherapy.

Liver function analysis demonstrated that all treatments increased the plasma AST and ALT levels, suggesting altered hepatocyte permeability. However, there was no change in the creatinine and urea levels, minimizing the risk of nephrotoxicity. Benznidazole toxicity is well-known.^62^ However, extrahepatic reactions are relevant side effects of this medicine.^11^ Furthermore, increased AST and ALT plasma levels are not invariably associated with manifestations of organic/systemic intolerance and should be monitored and maintained at acceptable levels during treatment.^78^ Current evidence indicates that the toxicity of the reference chemotherapy is dose-dependent,^56,66^ which also appears to occur with the combination of Bz and ME, since only the higher ME dose altered AST and ALT levels compared to Bz monotherapy. Despite the possibility of some degree of hepatotoxicity from ME + Bz-based chemotherapy, the cost-benefit ratio should be considered so that adjustments in the effective dose and treatment time can improve the therapeutic outcomes and the potential for parasitological cure, minimizing the risk of adverse effects for the infected host. This proposition should be pursued for ChD treatment using ME combined with Bz with a view to developing optimized antiparasitic protocols.

## Conclusions

Taken together, our findings indicate that ME exhibits physicochemical and ADME characteristics potentially compatible with orally bioactive drugs as well as direct antiparasitic effects on trypomastigote and amastigote *T. cruzi* forms, reducing parasitism and the parasite load of skeletal muscle cells. Similar to Bz, the antiparasitic effects of ME also manifested in vivo, especially when this drug was combined with Bz, resulting in prominent control of blood and skeletal muscle parasitism. These events are potentially linked to the myoprotective effects of the treatments, where the preservation of skeletal muscle microstructure also appears to depend on the attenuation of the inflammatory and oxidative muscle responses induced by Bz and ME, producing antiparasitic effects superior to monotherapy when combined.

## Abbreviations

ALT: alanine aminotransferase
AST: aspartate aminotransferase
ADME: absorption, distribution, metabolism, and excretion
AOPP: advanced oxidation protein products
BBB: blood–brain barrier
BZ: benznidazole
CBA: cytometric bead array
ChD: Chagas disease
CSA: cross-sectional area
DMSO: dimethyl sulfoxide
DNPH: 2,4-dinitrophenylhydrazine
ELISA: enzyme-linked immunosorbent assay
HE: hematoxylin and eosin
IFN-γ: interferon gamma
IgG: immunoglobulin G
IL-6: interleukin 6
IL-10: interleukin-10
LC–MS: liquid chromatography–mass spectrometry
LPO: lipid hydroperoxides
MDCK: Madin–Darby Canine Kidney cell
ME: 1-(2-(2-methoxy-6-nitro-4-propylphenoxy)­ethyl)-2-methyl-5-nitro-1*H*-imidazole
MPO: myeloperoxidase
NAG: *N*-acetyl-β-d-glucosaminidase
NFx: nifurtimox
NO: nitric oxide
PCN: protein carbonyl
PCR: polymerase chain reaction
qPCR: quantitative polymerase chain reaction
ROS: reactive oxygen species
RNS: reactive nitrogen species
R-*p*NP: *p*-nitrophenol derivative
TNF: tumor necrosis factor
TPSA: topological surface area
*V*_v_: volume density
